# Structural characterization of the *Streptococcus pneumoniae* carbohydrate substrate-binding protein SP0092

**DOI:** 10.1107/S2053230X16020252

**Published:** 2017-01-01

**Authors:** Simone Culurgioni, Minzhe Tang, Martin Austin Walsh

**Affiliations:** a Diamond Light Source, Harwell Science and Innovation Campus, Didcot OX11 0DE, England; b Research Complex at Harwell, Harwell Science and Innovation Campus, Didcot OX11 0FA, England

**Keywords:** *Streptococcus pneumoniae*, substrate-binding protein SP0092, ABC transporters, carbohydrate uptake

## Abstract

*Streptococcus pneumoniae* is an opportunistic respiratory pathogen that remains a major cause of morbidity and mortality globally, with infants and the elderly at the highest risk. *S. pneumoniae* relies entirely on carbohydrates as a source of carbon and dedicates a third of all uptake systems to carbohydrate import. The structure of the carbohydrate-free substrate-binding protein SP0092 at 1.61 Å resolution reveals it to belong to the newly proposed subclass G of substrate-binding proteins, with a ligand-binding pocket that is large enough to accommodate complex oligosaccharides. SP0092 is a dimer in solution and the crystal structure reveals a domain-swapped dimer with the monomer subunits in a closed conformation but in the absence of carbohydrate ligand. This closed conformation may be induced by dimer formation and could be used as a mechanism to regulate carbohydrate uptake.

## Abstract 2

The crystal structure of SP0092 was determined at 1.61 Å resolution and reveals a domain-swapped dimer with the monomer subunit in a closed conformation in the absence of ligand.

## 1. Introduction   

*Streptococcus pneumoniae* (the pneumococcus) resides asymptomatically in the upper airway tract but can migrate to normally sterile locations to cause diseases such as otitis, pneumonia, sepsis, septicaemia and meningitis (Weiser, 2010 ▸; Bogaert *et al.*, 2004 ▸). *S. pneumoniae* relies solely on carbohydrates as a source of carbon and, as these are limited in the nasopharynx, it dedicates over 30% of its transport systems to the uptake of carbohydrates, which are scavenged from host complex glycans (Burnaugh *et al.*, 2008 ▸; King, 2010 ▸; King *et al.*, 2006 ▸; Buckwalter & King, 2012 ▸; Bidossi *et al.*, 2012 ▸). These transport systems include phosphotransferase systems, ATP-binding cassette (ABC) transporters and porins, which provide the potential to convey up to 32 different carbo­hydrates (Bidossi *et al.*, 2012 ▸). In ABC transporters the ligand is translocated through the membrane by transmembrane permease domains activated by a pair of conserved cytoplasmic nucleotide-binding domains. In the case of type I and II ABC importers a substrate-binding protein (SBP) presents the bound substrate to the outward-facing side of the transporter, which selectively binds the ligand and transfers it to the transmembrane domains (Hopfner, 2016 ▸; Locher, 2016 ▸). SBPs are formed by two α/β domains connected by a hinge region, which are interdependent in the apo form (Tang *et al.*, 2007 ▸). Upon ligand binding at the interface between the two domains, the protein closes around the ligand in a more rigid conformation; ligand binding in this way has been termed the ‘Venus fly trap’ mechanism (Mao *et al.*, 1982 ▸). As the number of SBP structures determined has increased, the level of structural diversity has concomitantly grown. Six distinct structural groups have been proposed based on structural similarity, size and the presence of notable structural features (Berntsson *et al.*, 2010 ▸). This has recently been extended to a seventh structural class (G) following the structural characterization of FusA, a frucotoligosaccharide SBP from *S. pneumoniae* (Culurgioni *et al.*, 2016 ▸).

Here, we describe the crystal structure of the SBP SP0092 in an atypically closed and ligand-free conformation. SP0092 oligomerizes in solution in a concentration-dependent manner and we propose that dimerization could induce a closed conformation in which ligand binding is modulated. SP0092 belongs to the newly identified ‘cluster G’ structural subgroup class of SBPs, possessing an extended fold and large ligand-binding cavity that typifies this cluster.

## 2. Materials and methods   

### 2.1. Macromolecule production   

SP0092^39–491^ was cloned into the pOPINF vector (OPPF-UK), truncating the first 39 residues coding for the periplasmic localization signal. The native His-tag fusion protein was expressed in *Escherichia coli* BL21 Rosetta cells by autoinduction using Overnight Express medium (Millipore) supplemented with 1%(*v*/*v*) glycerol, while selenomethionine-labelled protein was expressed using SelenoMethionine Medium Complete (Molecular Dimensions) supplemented with 0.5 m*M* IPTG for induction. Cells were lysed in 0.1 *M* HEPES pH 7.5, 0.5 *M* NaCl, 0.02 *M* imidazole, 10%(*v*/*v*) glycerol supplemented with EDTA-free protease inhibitors (Roche) and cleared for 1 h at 100 000*g*. Cleared lysates were loaded onto an affinity HisTrap HP column (GE Healthcare). The fusion protein was eluted with lysis buffer supplemented with 0.2 *M* imidazole and, after dilution, was treated with HRV 3C protease overnight at 4°C. The mixture was loaded onto a HisTrap HP column and the cleaved protein was immediately eluted. The resulting sample was loaded onto a Superdex 200 column equilibrated with 0.02 *M* MES pH 6.5, 0.2 *M* NaCl, 2.5%(*v*/*v*) glycerol, 0.5 m*M* TCEP. Fractions of the two peaks observed from gel filtration were collected separately and concentrated to 170 and 154 mg ml^−1^ for the oligomeric and monomeric states, respectively. Macromolecule-production information is summarized in Table 1 ▸.

### 2.2. Size-exclusion chromatography and multiangle light scattering   

SP0092^39–491^ samples at different protein concentrations were loaded onto a Superdex 200 5/150 GL column equilibrated with running buffer [0.02 *M* HEPES pH 7.5, 0.2 *M* NaCl, 2.5%(*v*/*v*) glycerol, 0.5 m*M* TCEP]. Relevant collected fractions were loaded onto an SDS–PAGE gel. Static light-scattering experiments were performed at room temperature using a Superdex 200 Increase 10/300 GL column (GE Healthcare) in-line with a DAWN HELEOS II light-scattering detector (Wyatt). The column was equilibrated with running buffer. Samples of 100 µl protein solution at 5 mg ml^−1^ were analysed. Data acquisition and analysis were carried out using the *ASTRA* software.

### 2.3. Crystallization   

Initial crystals of SP0092^39–491^ were obtained by sitting-drop vapour diffusion at 20°C. These initial crystals were obtained by mixing equal volumes of protein (at a concentration of 50 mg ml^−1^) and a reservoir solution consisting of 20%(*w*/*v*) PEG 6000, 0.1 *M* Tris–HCl pH 8.0, 0.02 *M* zinc chloride. Optimization of the crystallization conditions resulted in single crystals of about 200 µm in size using the conditions detailed in Table 2 ▸. Selenomethionine-labelled SP0092^39–491^ yielded similar crystals in the same crystallization conditions.

### 2.4. Data collection and processing   

For data collection, crystals were first transferred to a cryoprotectant solution [reservoir buffer supplemented with 25%(*v*/*v*) glycerol] and then flash-cooled in liquid nitrogen. Crystal screening and initial crystal characterization were carried out on the I03 and I04 beamlines at Diamond Light Source. Diffraction data for selenomethionine-derivatized SP0092^39–491^ crystals were collected at the Se *K* edge. All data were processed with *xia*2 and resolution limits were defined using a half-data-set correlation coefficient (CC_1/2_) limit of 0.5, although the crystals diffracted to 1.48 Å resolution in the detector corners (Winter *et al.*, 2013 ▸). Data-collection and processing statistics are summarized in Table 3 ▸.

### 2.5. Structure solution and refinement   

The *SHELX* suite was used to determine the selenium substructure (Sheldrick, 2010 ▸). Analysis of the data with *SHELXC* showed a strong anomalous signal to high resolution, with a CC_1/2_ of 0.28 at 2.35 Å between observed and calculated *E* values (Schneider & Sheldrick, 2002 ▸). Data to 2.5 Å resolution (anomalous CC_1/2_ of 0.35) were used for the substructure search, which located all seven Se atoms. The atomic model was completed automatically with *ARP*/*wARP* with starting phases generated by *SHELXE*. The autotraced model was then completed through iterative cycles of manual model building and refinement using *REFMAC*5 in the *CCP*4 suite (Murshudov *et al.*, 2011 ▸; Langer *et al.*, 2008 ▸; Winn *et al.*, 2011 ▸) and *Coot* (Emsley *et al.*, 2010 ▸), respectively. The final refinement statistics are reported in Table 4 ▸. The final electron density was of high quality for the complete polypeptide chain except for the loop region formed by residues 90–96 (PDB entry 5mlt). The structure was visualized with *PyMOL* (http://www.schrodinger.com/pymol).

## 3. Results and discussion   

### 3.1. SP0092 oligomerization state   

**Figure 1:**
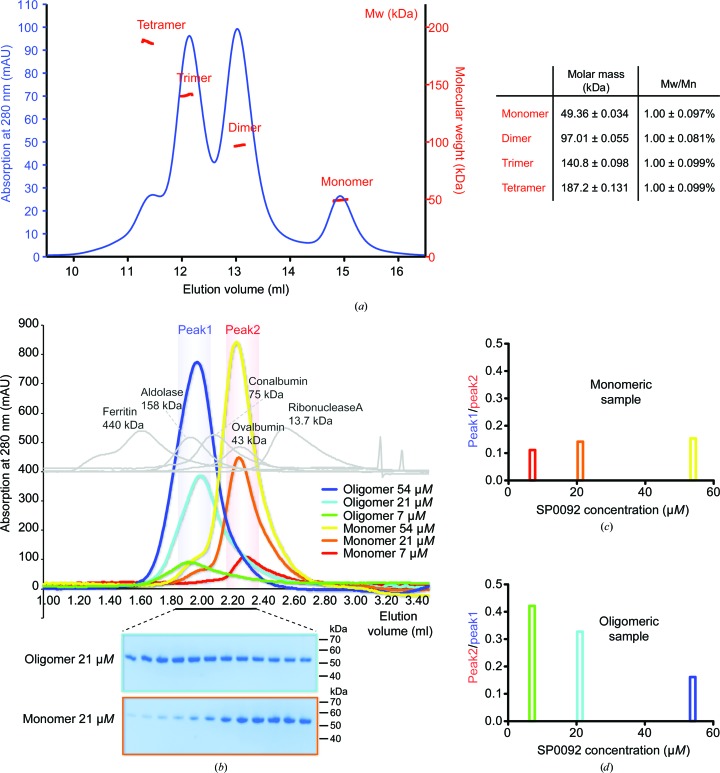
(*a*) SEC and multiangle light-scattering results for SP0092^39–491^. Absorption at 280 nm is shown in blue. The molecular weights of the different oligomerization states are shown in red. (*b*) SEC elution profiles of monomeric and oligomeric SP0092^39–491^ samples at different protein concentrations: monomer at 7, 21 and 54 µ*M* in red, orange and yellow, and oligomer at 7, 21 and 54 µ*M* in green, cyan and blue, respectively. SEC profiles of molecular-weight markers are shown in grey. SDS–PAGE of the elution fractions of oligomeric and monomeric samples at 21 µ*M* are shown in cyan and orange boxes, respectively. (*c*) The proportion of oligomeric state from the pooled monomeric sample was calculated as the ratio of the integrals of peak1 (1.9–2.1 ml) and peak2 (2.2–2.4 ml). (*d*) The monomeric state proportion of the oligomeric sample was calculated as the ratio of the integrals of peak2 and peak1.

Although the majority of SBPs are monomeric in solution, a few cases of higher order oligomerization states have been detailed (Schumacher *et al.*, 1994 ▸, 2004 ▸; Friedman *et al.*, 1995 ▸; Ramseier *et al.*, 1993 ▸). Following the observation of multiple elution peaks from size-exclusion chromatography, we measured the absolute molar mass of purified SP0092^39–491^ samples by multiangle light scattering (MALS). At least four different states were detected with good agreement to the theoretical molecular weights of SP0092^39–491^ monomer, dimer, trimer and tetramer species of 49.4, 97.0, 140.8 and 187.2 kDa, respectively (Fig. 1 ▸ *a*). To investigate whether the oligomerization is dependent on protein concentration, we analysed the gel-filtration elution profile of the monomeric and oligomeric samples at different dilutions. From this analysis, although the main species remained the same at different concentrations, we observed an increase in oligomerization of the monomeric sample at higher concentration (increasing from 10 to 13%); inversely, the monomeric state in the oligomeric sample increased from 14 to 30% of the total amount when diluted (Fig. 1 ▸ *b*). This points towards a dynamic equilibrium between the different species that is dependent on protein concentration (Figs. 1 ▸ *c* and 1 ▸ *d*).

### 3.2. Crystal structure of SP0092   

Both the monomeric and oligomeric species of SP0092^39–491^ isolated after size-exclusion chromatography were subjected to extensive crystallization trials, but only the latter yielded crystals and enabled the structure of oligomeric SP0092^39–491^ to be determined to 1.61 Å resolution (PDB entry 5mlt).

SP0092^39–491^ folds similarly to other substrate-binding proteins, presenting two globular α/β domains linked by a hinge region formed by three loops. The first domain (residues 39–154 and 321–396) is composed of one central β-sheet of four strands surrounded by seven α-helices, two 3_10_-helices and an additional three-stranded β-sheet. The second domain (residues 155–320 and 394–491) consists of a three-stranded β-sheet enclosed by eight α-helices, two 3_10_-helices and an extra three-stranded β-sheet.

**Figure 2:**
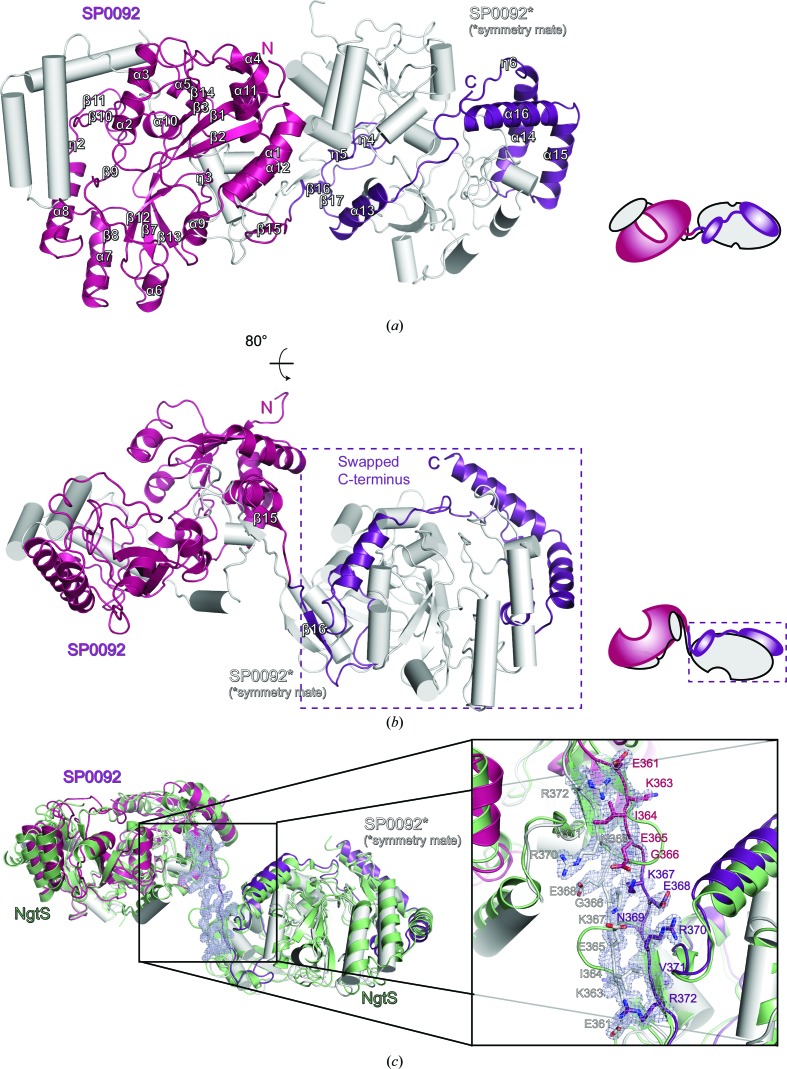
(*a*, *b*) Cartoon representation of the SP0092^39–491^ structure showing the swapped-domain dimer assembly in two orthogonal views. One protomer is coloured white and the other magenta (residues 39–366) and violet (residues 367–491). Each view is accompanied by a schematic representation of the swapped-domain dimer assembly. (*c*) Cartoon representation of a superposition of the SP0092^39–491^ dimer structure (magenta, violet and white) with the monomeric structure of SP0092, called NgtS (green; PDB entry 5suo). The electron-density map of the hinge loop connecting the swapped and main domains (2*mF*_o_ − *DF*_c_ at 1σ) is shown in blue/white mesh.

The most striking feature of the oligomeric SP0092^39–491^ structure is the presentation of a domain-swapped dimer structure. Crystals were only obtained from pooled samples of oligomeric SP0092^39–491^ and in the crystal asymmetric unit the ‘open’ monomer subunit of the swapped domain dimer extends its C-terminal domain (residues 367–491), positioning its β16 and β17 strands, its η4–η6 3_10_-helices and the last α13–α16 helices onto the neighbouring chain which is generated by the crystal symmetry (Figs. 2 ▸ *a* and 2 ▸ *b*). This domain swap generates an extended interface of 7550 Å^2^. The hinge loop connecting the swapped and main domains is located at residues Gly366 and Lys367, which are positioned between the β15 and β16 strands. The hinge loop is modelled in well defined electron density (Fig. 2 ▸ *c*). Apart from this hinge loop, the overall architecture of the two functional monomeric units is identical. A domain-swapped dimer structure has also been observed in the α-keto acid substrate-binding protein TakP (Gonin *et al.*, 2007 ▸). However, as of yet, there is no evidence that a domain-swapped dimer is a functional state of these SBPs.

### 3.3. Structural classification of SP0092   

**Figure 3:**
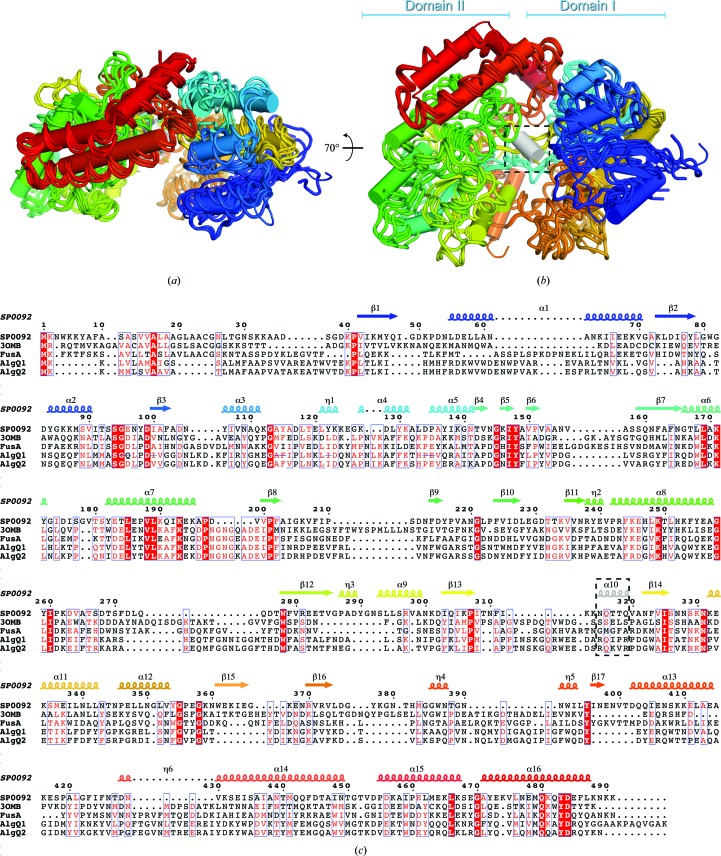
(*a*, *b*) Superposition of SP0092^39–491^ (functional monomeric unit), FusA (PDB entry 5g5y; Culurgioni *et al.*, 2016 ▸), AlgQ1 (PDB entry 1y3n; Momma *et al.*, 2005 ▸), AlgQ2 (PDB entry 1j1n; Mishima *et al.*, 2003 ▸) and Blon_2351 (PDB entry 3omb; Midwest Center for Structural Genomics, unpublished work) structures: the ribbon models, viewed in the indicated orientations, are coloured using rainbow colours (blue to red from the N-terminus to the C-­terminus). SP0092^39–491^ is shown with cylindrical helices. The dashed black rectangle highlights helix α10. (*c*) Sequence and secondary-structure alignment of SP0092, FusA, AlgQ1, AlgQ2 and Blon_2351 coloured according to their conservation using *ESPript* (Robert & Gouet, 2014 ▸).

The recent structure determination of the fructooligosaccharide substrate-binding protein FusA from *S. pneu­moniae* allowed a new subclass of SBPs to be defined. This structural subclass, annotated as subclass G, allowed the grouping of four SBP structures, including that of FusA. The members of subclass G are characterized by their larger molecular weight, additional structural elements, an enlarged ligand-binding cavity and a regulatory EF-hand-like calcium-binding site (Culurgioni *et al.*, 2016 ▸). SP0092^39–491^ possesses all of the features characterizing this subfamily apart from the calcium-binding site and shows approximately 24% sequence identity to the other subclass G members (Fig. 3 ▸). Independent structural superpositions of domains I and II, which make up the functional SP0092^39–491^ monomer, onto the equivalent domains of the other members of subclass G resulted in a maximum root-mean-square deviation of 2.92 Å for both domains of the monomers. The only prominent difference that is observed in the SP0092^39–491^ structure, when compared with the other subclass G members, is in the hinge region between the two α/β domains. In the case of SP0092^39–491^ the loop spanning residues 315–319 is reorganized to form an additional helix, α10. This helix is positioned in the central part of the ligand-binding cavity and may play a role in substrate interaction or recognition. Thus, in summary, we propose SP0092 to be a fifth, albeit atypical, member of the structural subclass G of SBPs.

### 3.4. Carbohydrate-binding cavity   

**Figure 4:**
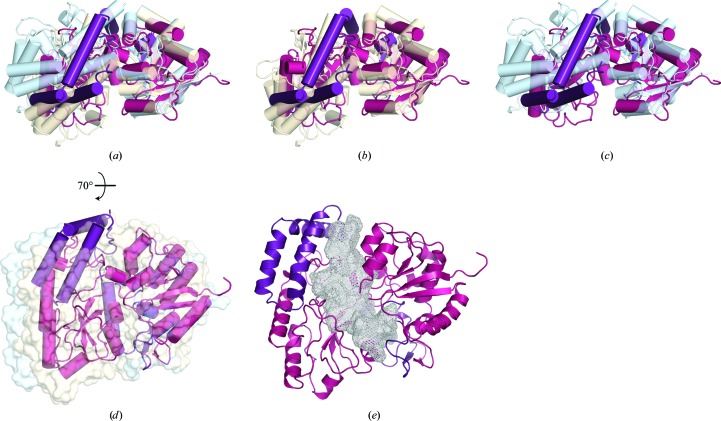
(*a*, *b*, *c*) Superposition of SP0092^39–491^ (functional monomeric unit; residues 39–366 in magenta and residues 367–491 in violet) with apo FusA (PDB entry 5g5y, light blue) and the FusA–nystose complex (PDB entry 5g60, light orange; Culurgioni *et al.*, 2016 ▸). The ribbon models are shown with cylindrical helices. (*d*) Superposition of SP0092^39–491^ (ribbon model with cylindrical helices in magenta and violet) with the surface representation of apo FusA (light blue) and the FusA–nystose complex (light orange). (*e*) Ribbon diagram of the structure of SP0092^39–491^ (magenta and violet), highlighting the substrate-binding cavity volume (grey mesh) obtained with *POCASA* (Zhang *et al.*, 2011 ▸).

Comparison of the SP0092 functional monomeric unit with the other members of subclass G reveals the subunit to be in a closed conformation even though no ligand is bound (Figs. 4 ▸ *a*–4 ▸ *d*). This may be a consequence of the domain-swapped dimer structure. Thus, variation in protein concentration may modulate ligand binding through the formation of a domain-swapped dimer, which presents a closed SBP monomer conformation.

Despite predictions for the binding of carbohydrates ranging from galactose, mannose and *N*-acetylmannosamine (ManNAc) by SP0092, the nature of the carbohydrate ligand still remains unknown (Bidossi *et al.*, 2012 ▸). The ligand-binding cavity of SP0092^39–491^ extends in volume to 2692 Å^3^, which is comparable to the closed ligand cavity of FusA (∼2218 Å^3^; Fig. 4 ▸ *e*). Thus, the structure of SP0092 shows that the SBP has the ability to bind complex oligosaccharides, which extend by at least three sugar moieties.

## 4. Closing remarks   

The pneumococcus relies solely on carbohydrates as a carbon source, with at least seven ABC transporters encoded in the reference genome strain TIGR4 annotated as carbohydrate importers. Here, we have determined the high-resolution crystal structure of the *S. pneumoniae* SBP SP0092, which delineates a large substrate-binding cavity and an overall structure which shows that it belongs to the newly described structural subclass G of the SBP family. Further structural analyses of the full complement of carbohydrate substrate-binding proteins could aid the investigation of these proteins as potential vaccine candidates and their potential suitability as novel drug-delivery systems (Saxena *et al.*, 2015 ▸; Garmory & Titball, 2004 ▸; Ahuja *et al.*, 2015 ▸).

*Note added in proof.* During the review of this paper, three entries were released by the PDB describing the SP0092 structure in a monomeric configuration with and without oligosaccharide bound (PDB entries 5swb, 5swa and 5suo).

## Supplementary Material

PDB reference: SP0092, 5mlt

## Figures and Tables

**Table 1:** Macromolecule-production information

Source organism	*S. pneumoniae* TIGR4
Forward primer	AAGTTCTGTTTCAGGGCCCGGACAAACCTGTTATCAAAATGTACCAAATCGGTG
Reverse primer	ATGGTCTAGAAAGCTTTATTTTTTGTTTTTCAAGAATTCATCGTATTGTTTTTGC
Expression vector	pOPINF
Expression host	*E. coli* BL21 (Rosetta)
Complete amino-acid sequence of the construct produced†	**GP**DKPVIKMYQIGDKPDNLDELLANANKIIEEKVGAKLDIQYLGWGDYGKKMSVITSSGENYDIAFADNYIVNAQKGAYADLTELYKKEGKDLYKALDPAYIKGNTVNGKIYAVPVAANVASSQNFAFNGTLLAKYGIDISGVTSYETLEPVLKQIKEKAPDVVPFAIGKVFIPSDNFDYPVANGLPFVIDLEGDTTKVVNRYEVPRFKEHLKTLHKFYEAGYIPKDVATSDTSFDLQQDTWFVREETVGPADYGNSLLSRVANKDIQIKPITNFIKKNQTTQVANFVISNNSKNKEKSMEILNLLNTNPELLNGLVYGPEGKNWEKIEGKENRVRVLDGYKGNTHMGGWNTGNNWILYINENVTDQQIENSKKELAEAKESPALGFIFNTDNVKSEISAIANTMQQFDTAINTGTVDPDKAIPELMEKLKSEGAYEKVLNEMQKQYDEFLKNKK

† The initial GP residues are the residual residues of the HRV 3C protease site.

**Table 2:** Crystallization

Method	Sitting-drop vapour diffusion
Plate type	96-well (Greiner)
Temperature (K)	293
Protein concentration (mg ml^−1^)	50
Buffer composition of protein solution	0.02 *M* MES pH 6.5, 0.2 *M* NaCl, 2.5%(*v*/*v*) glycerol, 0.5 m*M* TCEP
Composition of reservoir solution	18%(*w*/*v*) PEG 6000, 0.1 *M* Tris–HCl pH 8.0, 0.005 *M* zinc chloride
Volume and ratio of drop	100 nl protein solution and 100 nl reservoir solution

**Table 3:** Data processing and phasing statistics Values in parentheses are for the outer resolution shell.

Diffraction source	I04, Diamond Light Source
Wavelength (Å)	0.97950
Temperature (K)	100
Detector	PILATUS 6M
Crystal-to-detector distance (mm)	187.3
Rotation range per image (°)	0.15
Total rotation range (°)	360
Exposure time per image (s)	0.04
Space group	*C*2
*a*, *b*, *c* (Å)	102.24, 82.54, 60.35
α, β, γ (°)	90, 107.76, 90
Mosaicity (°)	0.22
Resolution range (Å)	49.64–1.61 (1.65–1.61)
Total No. of reflections	415088 (31945)
No. of unique reflections	62913 (4657)
Completeness (%)	99.9 (99.6)
Multiplicity	6.6 (6.9)
〈*I*/σ(*I*)〉	16.4 (4.0)
*R*_r.i.m._ (within *I*+/*I*−)	0.015 (0.277)
Overall *B* factor from Wilson plot (Å^2^)	20.0
Phasing	
Anomalous completeness	99.2 (100)
Anomalous multiplicity	3.3 (3.5)
CC_anom_ (from *AIMLESS*)	0.659 (0.059)
No. of selenium sites	7/7
FOM from *SHELXE* (2.5 Å)	0.66

**Table 4:** Refinement statistics for SP0092^39–491^ (PDB entry 5mlt)

Resolution range (Å)	49.64–1.61
Completeness (%)	99.94
σ Cutoff	None
No. of reflections, working set	59803
No. of reflections, test set	3158
Final *R*_cryst_	0.1947
Final *R*_free_	0.2287
Cruickshank DPI	0.0946
No. of non-H atoms	
Protein	3605
Ion	3
Water	33
Total	3946
R.m.s. deviations	
Bonds (Å)	0.01
Angles (°)	1.389
Average *B* factors (Å^2^)	
Protein	24.36
Ion	28.92
Water	33.60
Ramachandran plot	
Most favoured (%)	98.5
Allowed (%)	1.1
